# Antibody Responses against *Pneumocystis jirovecii* in Health Care Workers Over Time

**DOI:** 10.3201/eid1910.121836

**Published:** 2013-10

**Authors:** Serena Fong, Kieran R. Daly, Renuka Tipirneni, Leah G. Jarlsberg, Kpandja Djawe, Judy V. Koch, Alexandra Swartzman, Brenna Roth, Peter D. Walzer, Laurence Huang

**Affiliations:** San Francisco General Hospital/University of California, San Francisco, San Francisco, California, USA (S. Fong, R. Tipirneni, L.G. Jarlsberg, A. Swartzman, B. Roth, L. Huang);; Veterans Affairs Medical Center/University of Cincinnati, Cincinnati, Ohio, USA (K.R. Daly, K. Djawe, J.V. Koch, P.D. Walzer)

**Keywords:** Pneumocystis jirovecii, transmission, health care worker–patient, human immunodeficiency virus, HIV, major surface glycoprotein, pneumonia, respiratory diseases, fungi, HIV/AIDS and other retroviruses

## Abstract

In a previous cross-sectional study, we showed that clinical staff working in a hospital had significantly higher antibody levels than nonclinical staff to *Pneumocystis* *jirovecii*. We conducted a longitudinal study, described here, to determine whether occupation and self-reported exposure to a patient with *P. jirovecii* pneumonia were associated with antibody levels to *P*. *jirovecii* over time. Baseline and quarterly serum specimens were collected and analyzed by using an ELISA that targeted different variants of the *Pneumocystis* major surface glycoprotein (MsgA, MsgB, MsgC1, MsgC3, MsgC8, and MsgC9). Clinical staff had significantly higher estimated geometric mean antibody levels against MsgC1 and MsgC8 than did nonclinical staff over time. Significant differences were observed when we compared the change in antibody levels to the different MsgC variants for staff who were and were not exposed to *P. jirovecii* pneumonia–infected patients. MsgC variants may serve as indicators of exposure to *P.* *jirovecii* in immunocompetent persons.

*Pneumocystis jirovecii* pneumonia (PCP) is the leading AIDS-defining illness in the United States and is a serious complication in transplant recipients and other immunocompromised persons. Although understanding of the epidemiology and transmission of *Pneumocystis* spp. has increased, much remains unknown. Studies have demonstrated the ubiquity of *Pneumocystis* isolates in the environment and their presence in the human lung; however, little is known about the precise reservoir for the *Pneumocystis* species that infects humans (*P. jirovecii*) (*1*–*4*). Traditionally, PCP was thought to occur by reactivation of latent infection acquired during childhood, but more recent studies suggest that the disease can also occur after recent exposure and infection (*5*–*12*). Animal-to-animal airborne transmission of *Pneumocystis* organisms has been demonstrated after brief periods of exposure (*13*–*16*). These animal studies suggest that person-to-person airborne transmission can take place after brief periods of exposure.

The occurrence of PCP outbreaks in hospital and clinic settings supports the theory that *P*. *jirovecii* can be transmitted from a patient with PCP to an immunocompromised patient at risk for PCP (*17*,*18*). Studies have also demonstrated that immunocompetent hospital workers who care for patients with PCP can become colonized with *P*. *jirovecii* as can family members of PCP-infected patients (*19*,*20*). Animal studies have shown that immunocompetent mice become transiently colonized with *Pneumocystis* spp. after exposure to immunocompromised PCP-infected mice and that the colonized mice subsequently transmit and infect *Pneumocystis*-free immunocompromised mice (*21*). These findings suggest that colonized persons who do not have PCP may be another source of transmission.

In the absence of a culture method, seroepidemiologic studies have provided valuable insights into the epidemiology of *P*. *jirovecii* in humans. In our prior studies, we used an ELISA to measure IgG levels against the *P. jirovecii* major surface glycoprotein (Msg) (*22*,*23*). The ELISA identifies overlapping recombinant fragments that span the length of the Msg from the amino terminus (MsgA) to the middle portion (MsgB) to the carboxyl terminus (MsgC) (*22*). In an earlier cross-sectional study of hospital staff with clinical (with patient contact) and nonclinical (without patient contact) occupations, staff with clinical occupations had significantly higher serum antibody levels against the MsgC1 variant, but not against MsgA or MsgB, than did staff with nonclinical occupations (*23*). To examine antibody responses in these same health care workers over time, we conducted a longitudinal study of clinical and nonclinical hospital staff in which serial serum specimens were collected. These specimens were then analyzed for IgG against MsgA, MsgB, and MsgC1 and against 3 additional Msg constructs: MsgC3, MsgC8, and MsgC9. The study objectives were to determine whether clinical occupation and self-reported exposure to a patient with PCP were associated with higher antibody levels against the different Msg variants over time. Such findings would support the theory that patient-to-provider transmission of *Pneumocystis* isolates occurs in the hospital setting and address the use of antibody levels against Msg as epidemiologic markers of *Pneumocystis* infection.

## Methods

### Participants

A convenience sample of 115 San Francisco General Hospital (San Francisco, CA, USA) health care workers was enrolled in the longitudinal study from January 2007 through February 2009. HIV/AIDS Division and Division of Pulmonary and Critical Care Medicine staff were sought preferentially because they worked most consistently with patients who were infected with HIV and/or PCP, the presumed reservoirs of *P. jirovecii*. Recruitment was conducted primarily by word of mouth; emails were sent to departmental listservs; and announcements were made at staff meetings, medical conferences, and orientations for medical students and residents. At study entry, no respondents reported PCP infection. Participants who provided serial (2–5) serum samples were included in this analysis; these persons are a subset of the participants included in our prior cross-sectional study (*23*). The University of California, San Francisco, and the University of Cincinnati Institutional Review Boards approved this study. All participants provided written, informed consent.

### Questionnaires

Participants completed an initial baseline questionnaire at enrollment and a follow-up questionnaire every 4–8 weeks. Questions about demographic characteristics and medical history (i.e., cigarette smoking, chronic lung disease, and immunocompromising condition) were asked on the initial questionnaire. The questions regarding chronic lung disease and immunocompromising condition were purposefully generalized, and specification of any condition was made optional to protect confidentiality regarding a participant’s medical history. On both the initial and follow-up questionnaires, participants were asked whether they had ever been exposed to a patient with PCP and, if so, when their most recent exposure occurred. Participants were asked to choose from the following possible answers: within 1 hour, within 24 hours, within 7 days, within 1 month, and >1 month ago.

### Classification of Participants

Participants were placed in the following 2 categories: health care workers with patient contact and health care workers without patient contact, as described (*23*). Health care workers with patient contact (clinical staff) were persons who worked directly with patients in a clinical or research setting. Clinical staff included direct care providers, such as attending physicians, fellows, interns/residents, medical students, nurse practitioners, and nurses, as well as ancillary clinic staff, such as medical assistants, social workers, pharmacists, and clinical research assistants. Health care workers without patient contact (nonclinical staff) were persons who worked at the hospital but who did not work directly with patients in either a clinical or a research setting. Nonclinical staff included administrative and laboratory staff.

### Serum ELISA

Serum specimens were collected at the time of enrollment and quarterly (equal to a median of 3.2 months after the previous visit [interquartile range 3.0–3.5 months]) for up to 1 year, or for as long as each health care worker participated in the study. From each participant, 2–5 serum specimens were collected. All serum specimens were stored at −80°C and subsequently sent to the University of Cincinnati (Cincinnati, OH, USA) for analysis. An ELISA that included recombinant fragments derived from a single *P. jirovecii* Msg isoform was used to measure IgG levels (*22*). IgG responses against MsgA, MsgB, MsgC1, MsgC3, MsgC8, and MsgC9 were measured. All serum specimens from the same participant and a standard reference serum specimen were placed in duplicate wells in a 96-well plate and were tested against all 6 Msg fragments. Phosphate-buffered saline without antigen was used as a negative control. Reactivity was corrected by subtracting the reactivity of the serum to the phosphate-buffered saline from the reactivity of the serum to the antigen, and results were quantified by the method of Bishop and Kovacs (*24*). A standard reference serum specimen with specificity for each Msg construct was prepared by mixing serum from 4–6 specimens with high reactivity for the specific construct. These specimens were selected from testing banks of serum specimens from HIV-infected patients and healthy blood donors. From this, a standard curve was generated for each Msg construct on each day the assay was used. This curve was used to calculate the units of reactivity to the Msg construct. For each standard serum pool, we assigned a value of 100 units of reactivity to its target Msg construct in 100 μL of a 1:100 dilution. Test serum samples were diluted at 1:100–1:200 to fit the linear portion of the standard curves. Taking into account the dilution, we then calculated units of reactivity. The lowest possible value of 1 U was assigned to all specimens with values below the standard curve.

### Statistics

Demographic, health, and professional characteristics were compared by occupation (clinical vs. nonclinical). Simple categorical variables (sex, ethnicity, health conditions, exposure to PCP-infected patients) were compared by using the χ^2^ test. Fisher exact test was used when expected counts were <5. Multicategorical variables (race, work department) were compared by using logistic regression. Continuous variables (age) were compared by using the Student *t* test. Antibody levels were normalized by using a log transformation; results were exponentiated and presented as estimated geometric means (EGMs) with 95% CIs. Tobit mixed model regression for censored data was used to estimate the difference between antibody response in clinical staff and that in nonclinical staff. For a subset of workers who self-identified as having been exposed to a PCP-infected patient within 1 month before or after having a study serum specimen drawn, the changes in antibody levels from the time of exposure to 3 months and 6 months afterward were calculated and compared with changes from baseline to subsequent serum antibody levels in workers with no known *P. jirovecii* exposure. We compared antibody changes within each group using paired *t* tests and compared differences between the groups using a general linear model with 3-month or 6-month change as the dependent variable. Statistical significance was defined as p<0.05. All calculations were performed with SAS software 9.2 (SAS Institute Inc., Cary, NC, USA).

## Results

### Participants

We enrolled 115 staff members, and each staff member provided at least 2 serum specimens. Participants ranged from 22 to 80 years of age (mean 39.5 years), and 66 (57.4%) were female (Table 1). Seventy (60.9%) participants were White/Caucasian, 30 (26.1%) were Asian, and 3 (2.6%) were Black/African American. Seventeen (14.8%) were ethnically Hispanic/Latino. Thirty-nine (33.9%) participants had smoked at least 100 cigarettes in their lifetime; 19 (16.5%) had an underlying lung condition; and 8 (7.0%) had an immunocompromising condition. Fifty-two (45.2%) participants were part of the HIV/AIDS Division, 30 (26.1%) were part of the Division of Pulmonary and Critical Care Medicine (CCM), 27 (23.5%) were part of the Department of Medicine, and 6 (5.2%) were members of other departments (Obstetrics and Gynecology, Psychiatry, and Radiology). Of the 115 participants, 79 (68.7%) had a known exposure to a PCP-infected patient before the study period.

**Table 1:** Characteristics of San Francisco General Hospital staff in a study of antibody responses to *Pneumocystis jirovecii*, San Francisco, California, USA, 2007–2009*

Characteristic	Total no. (%), N = 115	Clinical, no. (%), n = 99	Nonclinical, no. (%), n = 16	p value
Demographic				
Mean age ± SD, y	39.5 ± 12.1	39.0 ± 12.4	42.8 ± 9.7	0.25
Sex				
F	66 (57.4)	56 (56.6)	10 (62.5)	Ref
M	49 (42.6)	43 (43.4)	6 (37.5)	0.66
Race:				
White/Caucasian	70 (60.9)	59 (59.6)	11 (68.8)	Ref
Asian	30 (26.1)	28 (28.3)	2 (12.5)	0.14
Black/African American	3 (2.6)	2 (2.0)	1 (6.3)	0.32
Multiple/other	12 (10.4)	10 (10.1)	2 (12.5)	0.95
Ethnicity				
Hispanic/Latino	17 (14.8)	13 (13.1)	4 (25.0)	0.25
Health condition				
Ever smoked	39 (33.9)	31 (31.3)	8 (50.0)	0.14
Lung condition	19 (16.5)	14 (14.1)	5 (31.3)	0.14
Immune condition	8 (7.0)	4 (4.0)	4 (25.0)	0.01
Professional				
Department/Division:				
HIV/AIDS	52 (45.2)	44 (44.4)	8 (50.0)	Ref
Pulmonary and Critical Care Medicine	30 (26.1)	27 (27.3)	3 (18.8)	0.49
Medicine	27 (23.5)	23 (23.2)	4 (25.0)	0.90
Other	6 (5.2)	5 (5.1)	1 (6.3)	0.81
Ever exposed to PCP patient	79 (68.7)	77 (77.8)	2 (12.5)	<0.001

*Ref., reference category; PCP, *Pneumocystis* *jirovecii* pneumonia.

### Participant Classification by Occupation

Ninety-nine (86.1%) participants were categorized as clinical staff, and 16 (13.9%) were categorized as nonclinical staff (Table 1). No significant differences were found between clinical and nonclinical staff in age, sex, race, ethnicity, smoking habits, underlying lung condition, or department/division. However, a significantly greater proportion of nonclinical staff than clinical staff reported having an immunocompromising condition (25.0% vs. 4.0%; p = 0.01). As expected, a significantly greater proportion of clinical staff than nonclinical staff reported exposure to a PCP-infected patient (77.8% vs. 12.5%; p<0.001).

### EGM Antibody Levels against Msg Over Time by Occupation

All staff had detectable antibodies against all Msg fragments: MsgA, MsgB, MsgC1, MsgC3, MsgC8, and MsgC9 (Table 2). For this study, the 99 clinical staff members provided a total of 396 serum specimens (mean 4 specimens), and the 16 nonclinical staff members provided 80 serum specimens (each provided 5 specimens). Overall, when levels of clinical and nonclinical staff were compared, clinical staff had significantly higher EGM antibody levels against MsgC1 (EGM 38.4 vs. 19.8; adjusted p = 0.004) and MsgC8 (EGM 46.0 vs. 27.6; adjusted p = 0.02), even when we controlled for age and immunocompromising condition. EGM antibody levels to MsgA, MsgB, MsgC3, and MsgC9 were not significantly different between clinical and nonclinical staff.

**Table 2:** Antibody levels against Msg, San Francisco General Hospital staff, San Francisco, California, USA, 2007–2009*†

Antibody	Occupation, estimated geometric mean (95% CI)			p value
	Clinical, n = 396	Nonclinical, n = 80		
MsgA	12.0 (11.1–12.9)	18.4 (17.1–19.9)		0.45
MsgB	4.9 (4.6–5.1)	6.7 (6.3–7.1)		0.56
MsgC1	38.4 (35.9–41.1)	19.8 (18.6–21.2)		0.004
MsgC3	77.1 (71.9–82.7)	52.9 (49.4–56.8)		0.09
MsgC8	46.0 (42.9–49.2)	27.6 (25.8–29.6)		0.02
MsgC9	34.6 (32.7–36.6)	27.0 (25.5–28.5)		0.17

*Msg, major surface glycoprotein. †By occupation for all time points and for all participants, adjusted for age and immune disorder,

### Change in EGM Antibody Levels against Msg Over Time in PCP-exposed and Never PCP-exposed Staff

**Figure 1:**
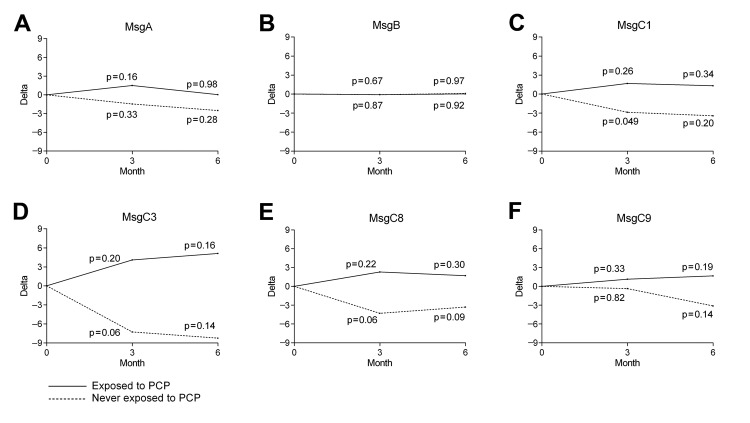
Differences in antibody levels against Msg at exposure to *Pneumocystis jirovecii* pneumonia (PCP) or baseline and 3 and 6 months later within groups of health care workers exposed and never exposed to PCP, San Francisco General Hospital, San Francisco, California, USA, 2007–2009. A) MsgA. B) MsgB. C) MsgC1. D) MsgC3. E) MsgC8. F) MsgC9. Msg, major surface glycoprotein.

To evaluate the relationship between PCP exposure and antibody response over time, we identified participants who reported being exposed to a PCP-infected patient within 1 month before any serum collection, irrespective of their occupation (exposed, n = 37), and compared their antibody levels at the time of exposure with their antibody levels 3 and 6 months later. As a control comparison, baseline serum antibody levels of never PCP-exposed persons (never exposed, n = 20), were compared with their antibody levels 3 and 6 months later. Thirty-three of the 37 (89%) exposed participants had been exposed to PCP-infected patients repeatedly over the study period. Members of the PCP-exposed group showed no significant difference in EGM antibody levels after PCP exposure for any Msg variant over time (Figure 1, panels A–F). In contrast to the findings in the PCP-exposed group, EGM antibody levels against MsgC1 in the never PCP-exposed group declined significantly after 3 months (mean change –2.87, 95% CI –5.74 to –0.01; p = 0.049) (Figure 1, panel C). Declines in EGM antibody levels against MsgC3 and MsgC8 over 3 months were of borderline significance (mean change –7.26, 95% CI –14.7 to 0.18; p = 0.06; and mean change –4.30, 95% CI –8.73 to 0.13; p = 0.06; respectively) (Figure 1, panels D, E). However, no significant differences were found in MsgA (Figure 1, panel A), MsgB (Figure 1, panel B), or MsgC9 (Figure 1, panel F) during this time.

**Figure 2:**
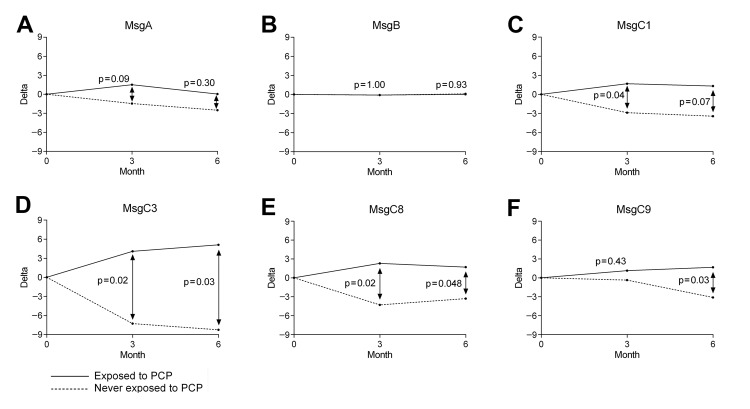
Differences in antibody levels against Msg at exposure to *Pneumocystis jirovecii* pneumonia (PCP) or baseline and 3 and 6 months later between groups of health care workers exposed and never exposed to PCP, San Francisco General Hospital, San Francisco, California, USA, 2007–2009. A) MsgA. B) MsgB. C) MsgC1. D) MsgC3. E) MsgC8. F) MsgC9. Msg, major surface glycoprotein.

Mean changes in EGM antibody levels within the PCP-exposed group were then compared with mean changes in the never PCP-exposed group (Figure 2, panels A–F). No difference was found in EGM antibody levels at baseline between exposed (antibodies measured at the time of exposure) and never-exposed (antibodies measured at the time of baseline enrollment) participants. In contrast, the difference in mean change was significant after 3 months for MsgC1 (mean change 1.67 vs. –2.87; p = 0.04) (Figure 2, panel C), after 3 and 6 months for MsgC3 (mean change 4.09 vs. –7.26, p = 0.02 and 5.10 vs. –8.24, p = 0.03, respectively) (Figure 2, panel D), after 3 and 6 months for MsgC8 (mean change 2.29 vs. –4.30, p = 0.02 and 1.71 vs. –3.30, p = 0.048, respectively) (Figure 2, panel E), and after 6 months for MsgC9 (mean change 1.67 vs. –3.11, p = 0.03) (Figure 2, panel F). After we adjusted for age and an immunocompromising condition, mean change after 6 months in MsgC1 became significant (mean change 1.31 vs. −3.43, p = 0.02). However, mean changes in MsgC3 and MsgC8 lost statistical significance. In contrast, no significant differences were found between the exposed and never-exposed groups in mean change for MsgA (mean change after 3 months 1.50 vs. –1.46, p = 0.09; mean change after 6 months 0.04 vs. –2.51, p = 0.30, respectively) (Figure 2, panel A), MsgB (mean change after 3 months –0.10 vs. –0.10, p = 1.00; mean change after 6 months 0.02 vs. 0.10, p = 0.93; respectively) (Figure 2, panel B), or MsgC9 after 3 months (mean change 1.14 vs. –0.35, p = 0.43) (Figure 2, panel F).

## Discussion

This study demonstrates that health care worker occupation is associated with antibody levels against *P. jirovecii* MsgC variants overall and that self-reported exposure to a PCP-infected patient is also associated with changes in antibody levels against MsgC variants over time. In our multivariate analysis, antibody levels to MsgC1 and MsgC8 were significantly higher for clinical staff than for nonclinical staff overall. Antibody levels to MsgC1, measured 3 months after baseline, had markedly declined in health care workers who were never exposed to PCP-infected patients, whereas those who were exposed to a PCP-infected patient showed no significant change in antibody levels 3 or 6 months after exposure. Significant differences were observed when we compared the change in antibody levels of all 4 MsgC variants for staff who were and were not exposed to PCP-infected patients. No significant associations were found between health care worker occupation and antibody levels to MsgA or MsgB. Also, no significant changes were found in antibody levels against MsgA or MsgB between the PCP-exposed and never PCP-exposed groups.

The carboxy terminus, or C fragment, is the most conserved region of the Msg (*25*), and IgG against MsgC1 and MsgC8 were significantly associated with clinical occupation overall. These findings are consistent with the results of our previous cross-sectional study, which found that MsgC1, but not MsgA or MsgB, antibody levels were significantly higher in clinical staff than in nonclinical staff (*23*). The current study also extends these findings to MsgC8, a variant that was not studied in the prior cross-sectional study. Previous studies involving HIV-infected patients with and without PCP infection showed that HIV-infected patients who also had PCP elicited greater responses against MsgC than did HIV-infected patients who did not have PCP (*26*). Djawe et al. found that PCP-infected patients had significantly higher serum antibody levels against MsgC1 at hospital admission, at 1–2 weeks, at 3–4 weeks, and at 5–6 weeks than did patients who had other types of pneumonia (*27*). Another study showed that patients who died of PCP had higher levels of MsgC8 than did those who died of other illnesses (*28*). A prior episode of PCP has also been shown to be a predictor of higher antibody levels against MsgC variants (*26*). These studies indicate that MsgC variants elicit significant host immune responses and antibodies against MsgC may serve as markers of infection in PCP-infected patients. Antibodies against MsgC may also be possible markers of exposure to and colonization with *P.* *jirovecii* in immunocompetent persons.

We observed no significant changes in antibody levels 3 or 6 months after exposure for health care workers who were exposed to PCP-infected patients. However, antibody levels against MsgC variants declined markedly in the never PCP-exposed group. When we compared the exposed and never exposed groups, we found a significant difference in mean change in antibody levels after 3 months against MsgC1, after 3 and 6 months against MsgC3, after 3 and 6 months against MsgC8, and after 6 months against MsgC9. Approximately 90% of the health care workers in the exposed group were repeatedly exposed to PCP-infected patients during the 6-month period. The lack of change in antibody levels against MsgC in the exposed group suggests that health care workers who are repeatedly exposed to *P*. *jirovecii* continue to mount an immune response to the organism. Participants in the never-exposed group may experience a gradual decline in antibody levels because of lack of exposure to *P*. *jirovecii*. These data support the theory that MsgC may be a marker of *Pneumocystis* exposure and colonization in persons who have contact with PCP-infected patients.

Our study had limitations, however. One limitation was that serum specimens were collected only every 3 months, which made it difficult to correlate serum antibody levels with specific periods of patient contact. Although no significant changes were observed in health care workers 3 or 6 months after exposure to a PCP patient, significant changes that occurred 3–4 or 5–6 weeks after exposure could have been missed. Future studies that collect serum specimens more frequently after exposure are needed to accurately measure the association between exposure to a PCP-infected patient and antibody development against *P. jirovecii*. Also, given the absence of published data to inform our study design, we decided at the start of this study to enroll a large number of representative health care workers who might be exposed to HIV-infected or PCP inpatients to explore whether antibody levels against Msg differed between clinical and nonclinical staff and between PCP-exposed and never PCP-exposed groups. Future studies that focus on collecting serial serum specimens from health care workers before and after PCP patient contact will further our understanding of *P. jirovecii* antibody production in healthy human adults. A second limitation was that questions related to *P. jirovecii* exposure on initial and follow-up questionnaires captured only information about the last contact with PCP-infected patients. The follow-up questionnaires were administered every 4–8 weeks, but the question about exposure accounted for contact that occurred only within the past 4 weeks. Thus, the precise time of an exposure was ambiguous. Future studies are needed that more accurately measure the interval, duration, and frequency of exposure to a PCP-infected patient.

Results from this study demonstrate that *P. jirovecii* elicits immune responses in health care workers who are exposed to PCP-infected patients and support the theory that *P. jirovecii* is transmitted from person to person in the hospital setting. Higher antibody levels seen over time in clinical staff than in nonclinical staff suggest that clinical staff, who are exposed to PCP-infected patients, may become subclinically infected with *P*. *jirovecii* and mount an immune response*.* Because animal studies have shown that immunocompetent mice exposed to PCP-infected mice can be carriers of *Pneumocystis* spp. and then transmit and infect immunocompromised mice, subclinically infected health care workers may thus be capable of transmitting the organism to immunocompromised patients. Future studies comparing Msg genetic sequences isolated from PCP-infected patients and the health care workers who care for them will further our understanding of *Pneumocystis* transmission. If the results from future studies support the theory of patient-to-provider transmission, respiratory precautions for PCP-infected patients may be necessary to prevent nosocomial transmission of *P*. *jirovecii*.
